# When Anger Strikes: Using AI Modelling to Understand How Negative Emotions Impact Performance in Digital Math Games

**DOI:** 10.3390/bs16040597

**Published:** 2026-04-17

**Authors:** Ana Zdravkovic Barber, Steve Engels, Earl Woodruff

**Affiliations:** 1 Department of Applied Psychology and Human Development (APHD), The Ontario Institute for Studies in Education (OISE), The University of Toronto, Toronto (UofT), Toronto, ON M5S 1V6, Canada; 2 Department of Computer Science, The University of Toronto (UofT), Toronto, ON M5S 1A1, Canada

**Keywords:** Digital Game-Based Learning Environment (DGBLE), affective dynamics, mathematics education, AI Modelling, AI emotion recognition

## Abstract

Digital game-based learning environments (DGBLEs) are increasingly integrated into classrooms as learning tools, yet limited research exists regarding the impact of students’ discrete emotions on digital gameplay performance. This study examined the role of emotions and arousal in predicting performance outcomes during digital gameplay. Thirty-two grade 5 students (*M*_age_ = 10.99, 62.5% male) played four digital games (two math; two identically designed non-math). During gameplay, real-time heart rate and affective data were collected and analyzed using an interpretable machine learning approach (XGBoost). Results suggest that students performed better on non-math games, as compared to math games. Real-time anger was associated with lower performance, particularly in games, whereas other emotions and physiological measures were not significant predictors. This pilot investigation suggests that discrete emotions, particularly anger, may play a more important role in performance during math gameplay than in comparable non-math activities. The results highlight the importance of supporting emotional regulation during digital math learning, as unmanaged anger may impact performance. This study contributes to the growing literature on affective dynamics in digital game-based learning.

## 1. Introduction

Emotions play a critical role in mathematics learning as they have the ability to impact attention, problem solving abilities, and perseverance (Pekrun, 2006; Abbot-Smith et al., 2023). Over-time, repeated, long-term experience of negative emotions during learning can impact engagement and interest in mathematics. Over the past two decades, researchers have long emphasized the role that math anxiety has taken in shaping students’ learning trajectories (Hembree, 1990; Luttenberger et al., 2018; Park et al., 2014; Ramirez et al., 2018), while growing evidence has indicated that moment-to-moment affective states may reveal more about learning ability, playing a critical role in shaping student success and approach to learning mathematics.

The widespread introduction of digital games for education offers a promising platform for learning that aims to combine engaging environments with educational topics to enrich learning for students (Mayer, 2019). Unlike traditional classroom instruction, digital learning environments can offer immersive contexts based on student interest (Boyle et al., 2012), provide students with opportunities to track their progress in real-time (Winnie, 2010), afford learners with immediate and ongoing feedback regarding performance (Shute et al., 2011), and even scaffold learning (Kiili et al., 2012). Research regarding the use of DGBLEs has primarily focused on achievement and engagement outcomes, with less attention on specific emotional processes that may unfold during gameplay. Among the studies that do measure affective responses during digital gameplay, the primary focus remains on positive emotions, such as enjoyment. Negative emotions experienced moment-to-moment during digital math learning remain under-researched, despite the well-established relationship between math performance and negative emotions in traditional learning methods (i.e., written tests; Zhang et al., 2019).

When implementing digital gaming, student knowledge regarding educational topics (i.e., math) can be assessed using either in-game or post-test assessments (Kiili & Ketamo, 2017). Many advancements in the field have allowed for real-time adaptations of games, where game developers and researchers have collaborated to understand student experience and performance based on multi-channel data (i.e., observation, self-report, physiological measurement). What is less understood is whether the data gathered in real-time during gameplay is attributed to the content (i.e., math topic or questions) or the actual game features (i.e., game design, ease of play). Without this type of information, it is unclear how emotions differ between math and non-math digital games, and how emotions may strongly influence learning and performance in digital contexts.

The present study examined whether discrete emotions, particularly anger, predict performance during math and non-math digital gameplay. Using a within-subjects design, students were asked to play two math games and two non-math games that were visually and structurally identical (i.e., matched game mechanics, interface, and progression), with mathematical content replaced by basic knowledge tasks (i.e., shape and colour identification). This design was purposeful, aiming to isolate the effect of math content while minimizing differences in gameplay complexity, although math tasks may have introduced additional cognitive load.

During gameplay, performance, physiological response (i.e., heart rate via Empatica E4; https://www.empatica.com (accessed on 21 March 2025), real-time facial emotions, and self-reported affect were collected. An explainable artificial intelligence (XAI) modelling approach, specifically an XGBoost regression model (T. Chen & Guestrin, 2016), was used to extract discrete emotional probabilities from Action Unit (AU) data obtained through post-processing video recordings using OpenFace 2.0 (version 2.2.0; Carnegie Mellon University, Pittsburgh, PA, USA; https://github.com/TadasBaltrusaitis/OpenFace (accessed on 20 March 2025); Baltrušaitis et al., 2018). Linear mixed-effects modelling (LMM) was used to account for repeated measures across gameplay trials (Meteyard & Davies, 2020).

### 1.1. Emotions and Math Learning

Mathematics is a foundational subject for future academic success; however, the affective experience while learning math impacts student persistence with the subject matter and how easily they can learn and can even predict future performance on math tasks (Geary, 2011). Marked by a feeling of tension and apprehension that then leads to avoidance and poor achievement, math anxiety is the most studied emotion related to math (Ashcraft & Kirk, 2001; Carey et al., 2016; Dowker et al., 2016). While over two decades of work has been dedicated to understanding the impact of math anxiety on performance, there is less understanding of discrete emotions, such as anger or enjoyment, and how they may impact math learning and performance.

Pekrun et al.’s (2006) Control-Value Theory of Academic Emotions provides a valuable framework for understanding how emotions impact learning. According to this theory, student’s affective experiences are a result of their perceived control over a task and how much value they assign it. For example, if a student feels that they are capable and good at math, viewing math as important to them, they are more likely to experience positive emotions, such as enjoyment. However, if a student does not feel like they have much control over the situation, for example if they believe the teacher’s test questions are unfairly difficult, or if they do not highly value the topic, negative emotions, such as frustration are likely to emerge. The experience of these emotions has been found to significantly impact learning by weakening students’ working-memory capacity, engagement, and persistence regarding the educational topic (Pekrun & Stephens, 2010; Pekrun et al., 2017; Loderer et al., 2020).

The experience of positive emotions during learning has consistently been found to improve educational performance and motivation. Students who experience greater instances of enjoyment and curiosity tend to show stronger engagement and problem-solving capabilities (Pekrun & Stephens, 2010). In contrast, negative emotions, such as anxiety, have disruptive impacts on learning, leading to disengagement and avoidance of learning topics (Ashcraft & Kirk, 2001). Further, the experience of boredom during learning, a commonly researched negative emotion, has been found to significantly reduce attentional capacity and engagement on specific tasks (Loderer et al., 2020). The experience of anger is less frequently studied in math education but may be quite impactful to learning as it is associated with frustration and difficulties with problem-solving (Pekrun et al., 2017; Riegel, 2021; Loderer et al., 2020).

To effectively capture students’ emotions, it is important to understand affective experiences as dynamic processes that unfold in real-time during learning. Students may shift between curiosity about a problem, frustration when the concept is tricky, and enjoyment when they move into a flow-state or effectively problem-solve (Loderer et al., 2020; Gutwin et al., 2016). Due to the ever-changing nature of emotions, it is critical to capture affective responses in real-time during learning activities, rather than relying solely on retrospective self-report data. Using multimodal data collection, researchers can effectively capture the dynamic emotional response individuals experience during learning using facial-video data and wearable technologies (Guedat-Bittighoffer et al., 2025; Liang et al., 2024). Despite the growing knowledge of sensor data for understanding affective responses, the majority of research conducted on negative emotions related to mathematics (i.e., primarily math anxiety) continues to rely on retrospective self-reports, failing to account for the impact of how “in-the-moment” affective experiences shape student engagement and performance during math learning.

### 1.2. Affective Responses During Digital Game-Based Learning

Digital game-based learning environments (DGBLEs) have become increasingly popular educational tools that promote engagement, motivation, and deep learning (Li et al., 2024; Su & Zou, 2024). The ability to embed academic content within interactive learning environments has afforded experiential situations that allow for concrete examples and learning by students (Clark et al., 2016; Ke, 2008). A recent meta-analysis by Clark et al. (2016), reported that the use of digital gaming environments can lead to improvements in achievement and motivation, as compared to traditional instructional methods (Wouters et al., 2013). Specific to math learning, the use of gamified learning environments has been associated with higher interest, persistence, and even deeper understanding of instructional topics (Ke, 2008), suggesting that digital games may potentially mitigate the experience of negative emotions during math learning. This benefit of digital learning environments may be due to the engaging presentation of topics in more concrete and meaningful ways to students, leading to increases in achievement and motivation for specific math topics (Beserra et al., 2014; Z. H. Chen et al., 2012; Rayya & Hamdi, 2001; Vogel et al., 2006).

The design of digital games for learning includes aspects that are challenging and may require feedback, meaning that students may experience failure or difficulty, which likely leads to the experience of negative emotions (Loderer et al., 2020). Recent research has noted that, depending on the task demands and outcomes, certain learning environments have been coined as “emotionally charged”, causing students to waver between positive and negative emotions (Plass & Kaplan, 2016; Piton et al., 2020). Thus, DGBLs emerge as data-rich environments that can harness real-time affective and physiological data measurement to better understand moment–moment affect and arousal during learning.

Another important feature of gamified environments is to include the ability for players to track and monitor their progress. This is done by including in-game features such as feedback systems and rewards. These mechanisms are critical for sustaining student engagement and motivation during digital gameplay. Specifically, by offering players a clear view of elements such as collected points, badges earned, and leaderboards, it allows individuals to not only track their progress and receive immediate feedback, but to experience a sense of accomplishment. This has been proven to enhance persistence and enjoyment during gameplay (Deterding et al., 2011; Hamari et al., 2014). Specifically, score displays and performance dashboards allow players to monitor their progress and set goals, while badges and achievement systems serve as extrinsic motivators that can reinforce effort and success (Domínguez et al., 2013). These game design principles are not only true for leisure games but have been found to extend into educational gaming environments in meaningful ways, resulting in deeper learning and engagement with the learning topic (Plass et al., 2015). When these in-game features are appropriately applied to digital gaming environments specifically designed for educational purposes, they can support learners’ positive affective experiences and maintain flow experiences during gameplay. Further, by providing timely feedback which can be depicted by the visible progress monitoring, these supportive scaffolding approaches can help to reduce negative affective experiences and optimize learning during challenging learning tasks (Xu et al., 2022), thereby improving performance through emotional regulation techniques. Similarly to traditional classroom environments, these digital learning environments provide a more powerful approach as compared to traditional teaching and evaluative methods. Namely, research conducted by Dicheva et al. (2015) found that by leveraging progress tracking, rewards, and feedback, digital games enhanced learners’ engagement and motivation. As such, digital learning tools stand to provide a significant enhancement in student performance and engagement with educational materials, more so than using traditional methods such as learning units and marks as the sole source of feedback.

Despite an influx of interest regarding technology-based environments for educational purposes, relatively few studies have focuses on the impact of discrete emotions on academic performance in math DGBLEs. Across the research, the focus remains on broad motivational outcomes, such as persistence, and positive emotional experiences, such as enjoyment (Hamari et al., 2016). Moreover, across the studies that do examine affective states during educational gameplay, most use self-report, limiting the understanding of the dynamic nature of emotions during learning (Mill et al., 2016; Pekrun, 2006). The current project addresses the gap in the literature by examining how discrete emotions, captured via a multimodal approach (i.e., real-time facial expression, self-report, and physiology), impact performance during digital math and identical non-math games. The project will provide insights into affective dynamics of learning and inform appropriate future design for emotional responsive technologies.

Emotions play a critical role in learning, influencing both attention and performance (Pekrun, 2006; Pekrun & Schutz, 2007), and digital games have been an effective tool for supporting these emotional and cognitive processes, offering interactive environments that promote motivation and engagement (Plass et al., 2015; Mayer, 2019), a critical gap in the literature remains regarding which aspects of performance are driven by challenges associated with mathematical topics rather than general in-game features. In order to address the current gaps in the literature, the current study examines the role of discrete emotional experiences during digital game-based learning and their impact on gameplay performance. In particular, this project focusses on whether real-time emotions, such as anger, emerge differently across math and non-math gameplay contexts and whether these emotional experiences are associated with performance outcomes.

The study is guided by the following research questions:

RQ1: Are there differences in gameplay performance between math and visually and structurally identical non-math digital games?
RQ2: Do students’ real-time emotional experiences (i.e., anger) predict gameplay performance across gameplay conditions?

Based on prior research, it is expected that students will demonstrate lower performance and more frequent negative emotional experiences during math gameplay, as compared to non-math gameplay. Negative emotions, specifically anger, are expected to emerge as significant predictors of performance outcomes during gameplay.

## 2. Methods

### 2.1. Participants

This study included thirty-two grade 5 students (*n* = 32, *M* = 11, *SD* = 0.42), 37.5% female, 62.5% male). A total of thirty-two primary caregivers completed a demographic questionnaire. According to caregiver responses, 78% (*n* = 25) of participants play educational digital games at home, while 21.8% (*n* = 7), do not. Meanwhile, 96.8% (*n* = 31) of participants are reported to play recreational digital games (i.e., Minecraft), whereas only 3.1% (*n* = 1) of participants reported not playing recreational digital games.

### 2.2. Procedure

A within-subject design was employed in which all participants completed four gameplay sessions: two math games and two non-math games that were visually and structurally identical (i.e., matched in game mechanics, interface, and progression), with mathematical content replaced by basic knowledge tasks (e.g., shape and colour identification). This design allowed for direct comparisons between gaming conditions, minimizing potential variability due to individual differences. Further, to reduce potential order effects, the games were presented in randomized order, ensuring that each participant experienced both math and non-math conditions of each game in varying order.

Throughout the study, participants wore an E4 wristband (Empatica E4 wristband; Empatica Inc., Boston, MA, USA; https://www.empatica.com; accessed on 21 March 2025) to record physiological responses. Prior to gameplay, participants completed the Achievement Emotions Questionnaire (AEQ-ES) to assess baseline academic emotions regarding math. During each gameplay session, real-time facial emotions were collected through video recording of the participants’ faces. Log-Trace Files were used to collect gameplay data (e.g., scores). Following each gameplay session, participants completed a post-game questionnaire assessing their emotional experience and gameplay perceptions. Time synchronized data from the E4 timestamps allowed precise alignment of physiological, behavioural, and self-reported data, enabling a detailed analysis of emotional responses across each gameplay condition.

### 2.3. Achievement Emotions Questionnaire-Elementary Students (AEQ-ES)

The Achievement Emotions Questionnaire-Elementary Students (AEQ-ES; see Appendix A) is a 28-item (nine enjoyment, 12 anxiety, and seven boredom items) self-report instrument used to assess elementary school students’ achievement emotions. It was adapted from the AEQ, a multidimensional self-report used to examine college students’ achievement and subsequent emotional experience in situations of academic achievement (Lichtenfeld et al., 2012). The questionnaire was adapted for use by elementary school-aged children and uses photos of both boys’ and girls’ faces to ensure both males and females could identify themselves. The instrument was designed to assess emotions of enjoyment, anxiety and boredom during mathematics in elementary school students. Enjoyment and anxiety were measured using three scales relating to experiences in the classroom, doing homework, and writing tests. Boredom was measured on two scales relating to experiences during class time and with homework. All items are answered on a 5-point Likert scale using these male and female faces, respectively (Lichtenfeld et al., 2012). The internal consistency for the AEQ-ES subscales has been demonstrated in prior research (Pekrun et al., 2011), with Cronbach’s α values ranging from 0.80 to 0.90 across enjoyment, anxiety, and boredom subscales. The AEQ-ES subscales demonstrated excellent internal consistency with regard to our dataset: enjoyment (α = 0.86), anxiety (α = 0.81), and boredom (α = 0.84).

### 2.4. Digital Learning Platforms

Two digital games, Space Chase and Factor Master, were developed by undergraduate computer science students at the University of Toronto. In order to compare the impact of math vs. non-math content (i.e., shapes/colours), a corresponding math-free version of each game was designed (Ore Chase and Block Master). Within these non-math games, math concepts were replaced with basic knowledge (e.g., shapes and colour identification). The math and non-math versions were designed to be visually and structurally identical, including matched game mechanics, interface, layout, gameplay progression, progress tracking and reward systems. This design approach was intentional, allowing for comparable gameplay complexity across conditions, such that the sole difference between the game versions was the presence or absence of math content. It is likely that the mathematics tasks imposed greater cognitive demands on participants, as compared to non-math tasks. Differences in performance and emotional response (i.e., increased frustration/anger) may reflect task difficulty, rather than math content alone. This should be considered when interpreting results.

The order of gameplay was randomized and counterbalanced across participants, such that each student was randomly assigned to begin either with the math or non-math version of each game. Each participant played all four games (two math-based and two non-math versions), in a randomized sequence.

#### 2.4.1. Game 1: Space Chase (Math Version of Game)

**Figure 1:**
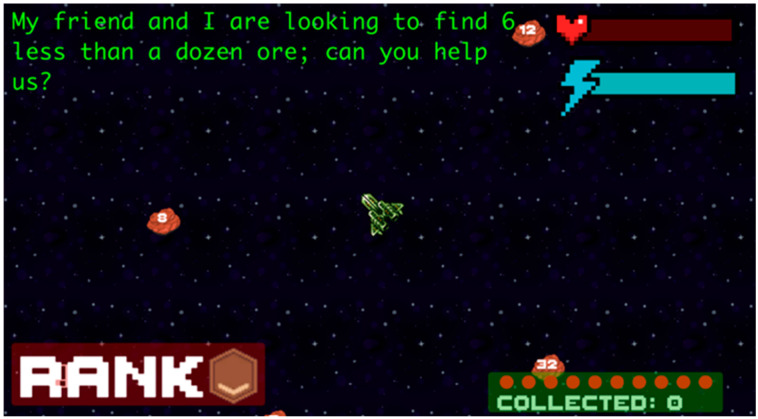
Space Chase math (math version of game).

Space Chase is a math word problem-based video game that integrates a playful, exploratory environment with mathematics word problems that test basic math fluency knowledge (see Figure 1). The game was developed to include grade-appropriate word problems that assess students’ knowledge of basic addition, subtraction, multiplication, and division. Each game begins with a tutorial that teaches students basic gameplay methods (i.e., use of arrow keys/click and drag mechanism). The player is required to move the spaceship around using the arrow keys to select the correct response. Students were able to see the number of correct responses by referring to the Collected icon in the bottom right. Based on the number of correct responses (i.e., number of collected) their rank would change from bronze, to silver, to gold, as seen in the bottom left of their screen. Log-Trace Data gathered automatically from the game was used to assess the number of correct and incorrect responses from each participant. Average scores were used to compare scores across participants.

#### 2.4.2. Game 2: Ore Chase Game (Non-Math Version of Game)

**Figure 2:**
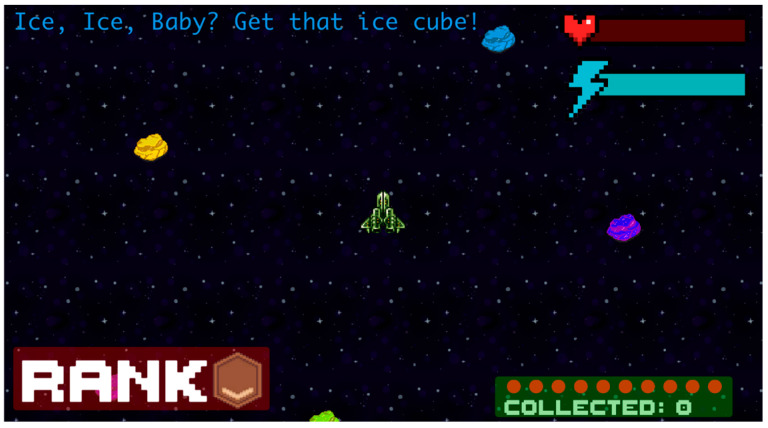
Ore Chase (non-math version of game).

Ore Chase is a word problem-based video game that was designed to be exactly like Space Chase Math, but without math features (see Figure 2). The game integrated a playful, exploratory environment with word problems that test basic colour knowledge. The participant was required to move the spaceship around using the arrow keys to select the correct response. Students were able to see the number of correct responses by referring to the Collected icon in the bottom right. Based on the number of correct responses (i.e., number of collected) their rank would change from bronze, to silver, to gold, as seen in the bottom left of their screen. Log Data gathered automatically from the game was used to assess the number of correct and incorrect responses from each participant. Average scores were used to compare scores across participants and games.

#### 2.4.3. Game 3: Factor Master (Math Version of Game)

**Figure 3:**
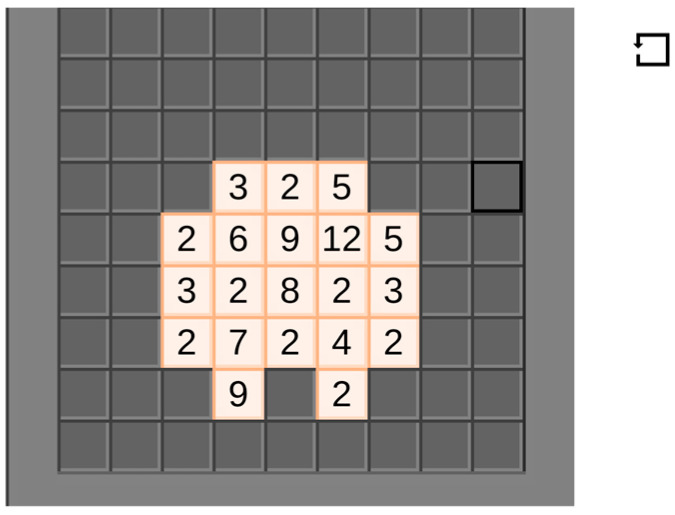
Factor Master game (math version of game).

**Figure 4:**
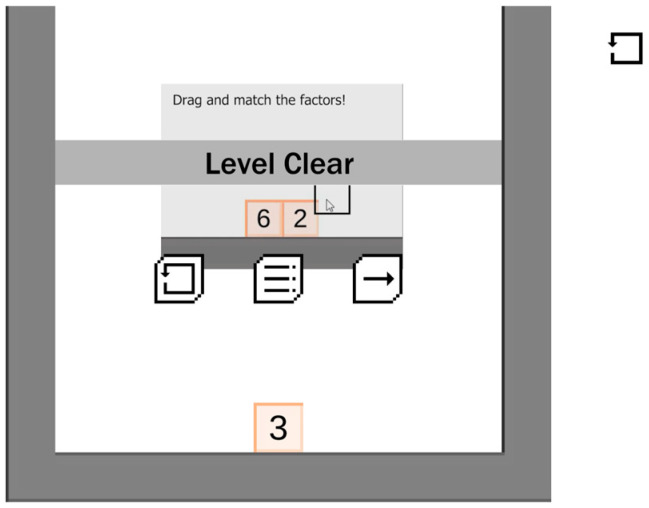
Factor Master game progress monitoring.

Factor-Master is a game that tests basic knowledge of factors and factoring numbers (see Figure 3). The game includes a set of trial/instructional stages that teach the player how to navigate the gaming environment and how to select correct answers. The game integrates a fun block-like background with numbers on each block. The participant is required to drag and select all numbers that are factors and their component factors or multiples. Once the blocks are correctly selected, they disappear, and the student was able to track their progress when given the information about level progression (see Figure 4). Log Data gathered automatically from the game was used to assess the number of correct and incorrect responses from each participant. Average scores were used to compare scores across participants and games.

#### 2.4.4. Game 4: Block Master (Non-Math Version of Game)

**Figure 5:**
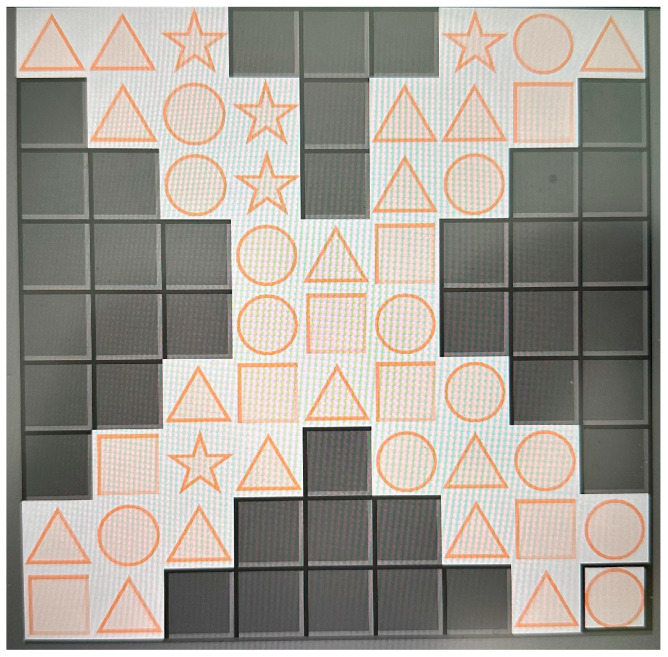
Block Master game (non-math version).

Block master is a game that tests basic knowledge of shapes (see, Figure 5). The game was designed to include a set of trial/instructional stages that teach the player how to navigate the gaming environment and how to select correct answers. The game integrates a fun block-like background with shapes on each block. The participant is required to drag and select blocks that have the same shape on them (i.e., triangles, circles, squares). Once a group of blocks are correctly selected, they disappear, and once the full screen is cleared, the players progress to the next level of the game, as depicted with the progress monitor (Figure 4). Log Data gathered automatically from the game was used to assess the number of correct and incorrect responses from each participant. Average scores were used to compare scores across participants and games.

### 2.5. OpenFace Software

OpenFace 2.0 (version 2.2.0; Carnegie Mellon University, Pittsburgh, PA, USA; https://github.com/TadasBaltrusaitis/OpenFace, accessed on 20 March 2025; Baltrušaitis et al., 2018) is an open-source toolkit for facial expression coding that was used to measure emotions on the face during gaming. The software uses the Facial Action Coding System (FACS; Ekman et al., 2013). Pre-recorded video data was processed through OpenFace to measure musculature movement in the face at 18 different Action Units (AUs) and one blink (AU45) to code into scores of the eight basic emotions (happiness, sadness, surprise, fear, anger, disgust, contempt and neutral). The software has been widely used in the literature and validated for research with both children and adults (Aydın & Özyazıcıoğlu, 2023), including the assessment of postoperative pain in children with computer-assisted facial expression analysis. The output of AU intensity and presence is provided by OpenFace software, and can be used to classify emotions using machine learning techniques.

### 2.6. Machine Learning Techniques for Extracting Emotion Labels

Supervised machine learning was implemented to identify patterns of AUs associated with each emotion. Each video was decomposed into individual frames to allow for detailed facial analysis. Frames were then processed through a multi-step pipeline to detect and quantify facial expressions associated with emotion. Action Units (AUs), representing discrete facial muscle movements, were extracted from the spatial configuration of landmarks. Videos were from three publicly available datasets including MMI, Oulu-Casia and DDCF. Each video was processed using a multi-stage approach involving face detection, facial pose estimation, landmark detection, and Action Unit (AU) extraction. The resulting AU sequences were analyzed to detect emotions. Videos were pre-processed and decomposed into individual frames and MobileFaceNet (S. Chen et al., 2018) was used to provide landmarks that served as the detection for muscle activation. The AU vectors provided a quantifiable representation of facial activity that is relevant for emotion recognition. AU sequences were input into a machine learning model designed to classify dynamic facial expressions into seven discrete emotions. An Extreme Gradient Boosting (XGBoost; J. H. Friedman, 2001; J. Friedman et al., 2000) framework was trained to map landmark positions to AU values. This model learned the temporal progression of facial movements across frames, rather than analyzing frames independently, enabling richer detection of emotional states. For each video, detected emotions were timestamped according to frame position and arranged in chronological order. These sequences facilitated visual and quantitative analyses of emotional progression throughout gameplay. All computations were performed using Python (version 3.10; Python Software Foundation, Wilmington, DE, USA; https://www.python.org, accessed on 20 March 2025; Python Software Foundation, 2022)

Each emotion was associated with a combination of AUs, with thresholds set to determine the presence of these AUs in each frame (Table 1). Thresholds were determined based on established guidelines from previous research. Baseline measurements for each AU were calculated per participant and peaks of each emotion were identified by comparing real-time AU intensities to the established baseline (Li et al., 2024). Analyses were conducted to compare the frequency and intensity of emotional peaks across different conditions (i.e., across each gameplay session).

**Figure 6:**
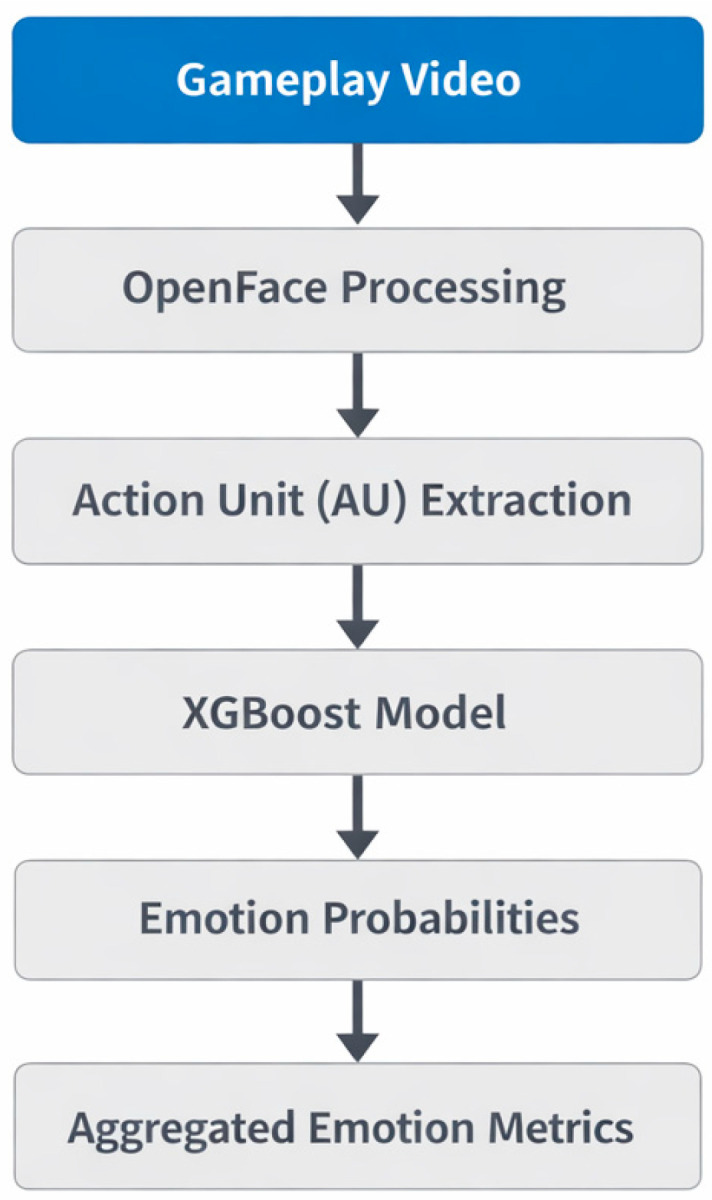
Emotion recognition pipeline flowchart.

Once trained, the model was used to extract affective sequences from the raw data collected for this project (i.e., math vs. non-math gameplay). Each video was compared using the above metrics that provided frequency, intensity and duration of each emotion. A spike in negative emotions was defined using intensity and duration thresholds, consistent with prior work demonstrating that peak emotional moments disproportionately influence overall experience (Kahneman et al., 1993; Gutwin et al., 2016). Any instances and sequences exceeding thresholds were included as spikes. Frequency analyses quantified the number of negative emotion spikes per video and compared averages across gameplay videos. The XGBoost model’s performance was evaluated using cross-validation on a held-out subset of frames, achieving moderate classification accuracy across the seven emotions (e.g., average F1 score = 0.65). Although performance was sufficient for extracting general affective trends, further improvement is possible with larger datasets or more advanced architectures. Given the modest size of the gameplay video dataset, the study was likely underpowered to detect small differences in less frequent emotional expressions. The overall emotion recognition pipeline is illustrated in Figure 6.

### 2.7. Heart Rate Data

**Figure 7:**
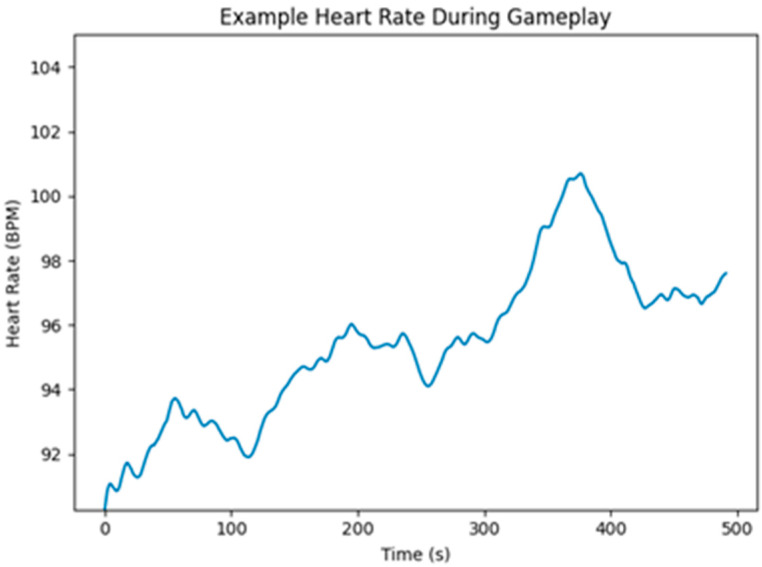
Example heart rate during gameplay. Note: This illustrates (HR) data collected during a gameplay session for a single participant. Initial signal artefacts were removed. The *Y*-axis is scaled to include the full range of observed values, illustrating natural fluctuations in heart rate across gameplay. Time is represented in seconds.

Heart rate (HR) data was sampled in real time using the Empatica E4 wristband (Empatica Inc., Boston, MA, USA; https://www.empatica.com; accessed on 21 March 2025). All HR data gathered from the E4 was preprocessed using MATLAB (version 9.13.0; The MathWorks, Inc., Natick, MA, USA; https://www.mathworks.com, accessed on 21 March 2025; The MathWorks, 2022) and Ledalab (version 3.4.9; http://www.ledalab.de/, accessed on 21 March 2025; Benedek & Kaernbach, 2010a, 2010b; Posada-Quintero & Chon, 2020) Raw HR signals were inspected for artefacts (i.e., motion/poor sensor contact/noise/outliers) and any missing segments were excluded. Cleaned HR data was used to compute average HR and HR variability (HRV). Data was then exported and organized by participants for analysis. An example of heart rate data captured during gameplay is presented in Figure 7.

## 3. Results

A Linear Mixed-Effects Model (LMM) was conducted using RStudio (version 2023.06; RStudio PBC, Boston, MA, USA; https://posit.co, accessed on 20 March 2025; RStudio Team, 2023) to examine the effects of game type (math vs. non-math) and participant emotions on gameplay performance outcomes. A random intercept for subjects was included to account for repeated measures design. An initial model including a random slope for game type by subject produced a singular fit, indicating that there is insufficient data to estimate these effects reliably by subject. Results indicated that game type (i.e., math vs. non-math) significantly predicted performance, where participants scored higher on non-math games (*M* = 84.7, *SD* = 13.0) as compared to math games (*M* = 58.3, *SD* = 13.4), *b* = 26.80, *SE* = 2.52, *t*(127) = 10.62, and *p* < 0.001. The effect is strong, and we can say so with good certainty.

When including all self-report and sensor data (heart rate, facial emotions, and AEQ scores), anger, as measured in real time, emerged as a significant negative predictor of performance (*b* = −15.24, *SE* = 5.67, *t*(127) = −2.69, *p* = 0.008), such that higher anger was associated with lower scores. This effect is moderately strong and suggests a meaningful negative impact of anger on learning outcomes, particularly in math contexts.

All other predictors, including heart rate (β = 1.84, *SE* = 1.38, 95% CI [−0.87, 4.55], *p* = 0.18), happiness (β = 22.99, *SE* = 21.67, 95% CI [−19.47, 65.46], *p* = 0.29), and self-reported emotions (enjoyment, anxiety, boredom), were nonsignificant, with wide confidence intervals that included zero. The effects of these predictors are almost zero, and we can say so with reasonable certainty (Meteyard & Davies, 2020).

**Figure 8:**
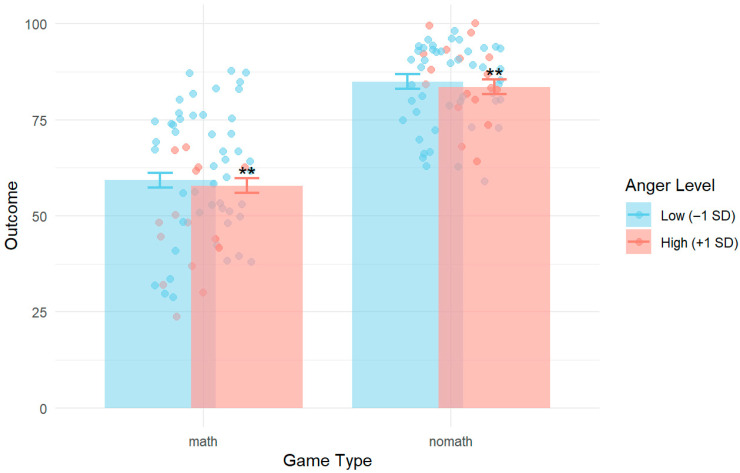
Model-predicted and observed outcomes by game type and anger. *Note:* Error bars represent standard errors. Anger levels reflect ±1 SD from the mean. ** *p* < 0.01 indicates a significant effect of anger on performance.

The random intercept variance for subjects was 21.7 (*SD* = 4.66), and the residual variance was 170.0 (*SD* = 13.04), indicating substantial between-subject variability alongside within-subject variability. A simplified model including only game type and anger confirmed these effects. Findings indicated that high anger reduced performance in both math and non-math games, while performance on non-math games was consistently higher than math-games. These results suggest that while game type strongly influences performance, anger during gameplay also plays a meaningful role in student outcomes, highlighting the importance of emotional regulation in math learning contexts (Table 2 and Table 3 and Figure 8).

## 4. Discussion

The current study examined the impact of affective response and physiological features on performance in math and non-math digital gaming environments. Regarding our first research question, which examined differences in performance between math and non-math gameplay, student performance was consistently higher on non-math games, as compared to visually identical math games. This finding illustrates the added cognitive demands related to answering math questions, compared to accessing simple non-math knowledge (i.e., colours/shapes). Research question two explored whether in-game emotional experiences predict gameplay performance. Results indicated that real-time experiences of anger during gameplay emerged as a significant negative predictor of performance, with a particularly strong effect on math-specific digital games, as compared to identical non-math games. Conversely, other real-time emotions (i.e., happiness/sadness), and heart rate measurements were not found to be significant. The results highlight that the experience of anger during learning may play a larger role in shaping student academic performance in educational gaming contexts. These findings highlight that the experience of anger during learning may impact student academic performance to a larger extent than other affective experiences, specifically within gaming contexts. The results are aligned with previous research, namely one longitudinal study performed by Forsblom et al. (2022), which demonstrated that anger negatively predicts subsequent math achievement over time. This project adds a novel approach to understanding the impact of anger by implementing a real-time data capture methodology using video recordings of students’ faces during gameplay, rather than solely relying on self-reported data to capture student emotional responses. Further, this project adds to the literature by designing and assessing performance and affect during digital gameplay.

The findings that real-time emotions such as happiness and sadness, as well as heart rate measures, were not significant predictors of performance may reflect the complexity involved in mapping physiological and affective processes onto discrete emotions. While heart rate is commonly associated with arousal, it is not a reliable indicator of specific emotional experiences, especially within tasks that are short in length, such as the gameplay sessions used in this research project. Rather, physiological signals, such as heart rate, may indicate general arousal or engagement in tasks. Most emotions, such as happiness and sadness were not significantly elicited during gameplay, while anger-related facial expressions may be related to task difficulty or frustration with task performance in digital learning environments. These findings are aligned with previous research which suggests that physiological arousal measures do not always map directly onto discrete emotional categories, especially in real-world learning contexts (Barrett, 2006; D’mello & Kory, 2015). Although anger emerged as a significant predictor of performance in this research project, it is important to note that the experience of other emotions (i.e., enjoyment, confusion) may also play a large role in shaping student performance and learning within digital game-based learning and should be explored in future research.

The results of this project highlight the importance of affective response when designing digital gaming environments for educational purposes. The inclusion of features, such as points, badges, progress monitoring, and adaptive feedback are commonly associated with digital learning environments and have been shown in prior research to enhance motivation and persistence by outlining clear goals, providing immediate feedback and leaving students with a sense of accomplishment during gameplay (Deterding et al., 2011; Hamari et al., 2014; Plass et al., 2015). Research by Domínguez et al. (2013) similarly illustrated that providing achievement systems and badges enhances learners’ motivation and leads to sustained effort. It is important to note, however, that the present study did not directly measure these game features in isolation. As such, the findings denote that negative emotional responses, specifically anger, may play an important role in shaping student performance during gameplay. Within these digital learning contexts, features such as immediate feedback or adaptive difficulty may be important tools for mitigating frustration and fostering engagement, though this warrants further investigation and research. In the context of math learning, where anger was found to negatively predict performance, incorporating real-time affective feedback and emotional regulation strategies (e.g., calming prompts or adaptive difficulty) may help sustain engagement and learning outcomes (Shaheen et al., 2023).

It is noteworthy that both versions of each game were designed to be visually and structurally identical, other than the specific math questions included in the math versions. This was done to ensure that the specific design features (i.e., progress monitoring, levels, etc.) were not responsible for differences in negative emotions and in-game scores. These findings suggest that math content may contribute to elevated anger and reduced performance; however, additional controls (i.e., previous math proficiency) are needed to confirm this interpretation. This study draws on Gee’s (2023) perspective on the learning potential of video games; however, it is important to acknowledge that the relationship between gameplay and emotional or learning outcomes is not inherently linear. Digital game design, mechanics, and cognitive demands vary immensely, and as such, may elicit a range of emotional responses depending on the specific learning task and context. The present findings highlight that educational gameplay for mathematics can also evoke negative emotions such as frustration or anger, which may influence performance. As such, digital game-based learning environments should be examined not only as motivating tools, but as complex learning environments in which both positive and negative affective experiences emerge. As a pilot study, the findings from this study should be interpreted as preliminary and intended to guide future research.

The findings from this project have implications for not only research, but also educational practice. The implementation of emotional regulation programming during mathematics learning is imperative, as negative affect was a significant predictor of performance. Specifically, the finding that anger, as measured in real time by analyzing facial-emotions data, suggests that the experience of negative emotions related to difficult mathematical tasks depletes their cognitive resources needed for problem-solving and redirects them towards managing their experience of anger. This aligns with Control-Value Theory (Pekrun, 2006), which posits that discrete emotions directly impact engagement and achievement, with negative activating emotions like anger significantly impairing the learning processes. These results highlight the impetus for designing specific educational technologies and AI tutors (Woodruff, 2025) geared towards, not only cognitive and academic skill development, but which also provide clear approaches that include scaffolding to support emotional regulation skills. By integrating tools such as real-time emotion detection during gameplay, these DGBLEs could thus become adaptive tutoring systems that respond to student affective states during learning, help to sustain motivation, and even mitigate the experience of negative emotions (i.e., frustration, anger) during math learning.

## 5. Limitations and Future Directions

This study is not without its limitations. A key limitation is the absence of controls for prior math ability, including students’ baseline math proficiency, prior achievement levels, or familiarity with the specific math topic. This is significantly important as the findings of our study highlight clear differences in performance between math and non-math games and this is potentially influenced by individual differences in math ability as opposed to math condition alone. Further, students with lower ability and higher math anxiety may have been more susceptible to increases in frustration or anger during gameplay, thereby contributing to the observed relationship between affect and performance on math games. Interpretations of these findings should be made with caution, and future research should incorporate baseline measurements of math ability to form a stronger understanding of the impact of emotional experiences on performance. Additionally, this study only included two game types, each with corresponding non-math versions, which may limit the generalizability of the findings. While the games were designed to be visually and structurally identical, there is a possibility that certain features within the games impacted both emotional responses and performance. As such, it is difficult to discern whether observed differences are due to math content or in-game characteristics of the specific games. This limitation is particularly relevant when interpreting the impact of anger on math gameplay. Future research should assess a larger number of digital games with differing math tasks to better isolate the role of content as compared to design features. This would enhance the generalizability of findings across digital learning contexts and math concepts.

A second limitation of our study was the relatively small sample size (*N* = 32), which may impact the generalizability of findings and may limit the statistical power for detecting more nuanced implications emotions have on performance within gameplay. Further, this sample included only grade 5 students, which impacts the ability to extend the conclusions from our results to learners of differing ages. In terms of game inclusion, it would extend the findings of this study if more games were included for analyses, as using two different games may not capture the full range of in-game features that may be impacting affect and performance.

A further limitation of this project is the accuracy of the emotion detection pipeline used to estimate students’ affective states from facial expressions. While the Action Unit (AU)-based approach and ML models were trained on established benchmark datasets, there are risks when applying any automatic emotion detection software to naturalistic datasets, namely regarding potential misclassification. This occurs due to lighting variability, head movement, occlusion, and individual differences in facial expressions. As such, the accuracy of AU detection and emotion classification has the potential to be reduced, especially when classifying discrete emotions (i.e., happiness, anger), and may fail to capture more complex internal affective experiences. The primary findings of our study rely specifically on this emotion detection approach, denoting anger as a significant predictor of performance. As such, any potential inaccuracies in emotion classification may impact results and interpretations. Findings should be interpreted with caution, given previously highlighted challenges with subtle emotion detection in real-world scenarios (Barrett et al., 2019; D’mello & Kory, 2015). Despite the strengths of real-time emotion detection, they may fail to capture the full depth of emotional experience, as many affective processes are internal and not directly observable. Moreover, as children develop, they increasingly learn to regulate, mask, or strategically conceal emotional displays, which can further limit the validity of observational measures (Saarni, 1989). While our research did include multiple measurements of affective components (i.e., real-time facial emotions, self-report, HR) to control for this, the project did not account or control for the impact of contextual features, such as prior math knowledge, mastery of specific math topics or math grade-level achievement, misclassification of FACS, the absence of prior math proficiency control and possible item difficulty mismatch.

A related limitation is regarding the generalizability of the machine learning model used for emotion detection. Our model was trained on multiple benchmark datasets, including those containing children’s faces (e.g., DDCF), variable facial expressions (e.g., Oulu-CASIA), and prototypical laboratory-based datasets (e.g., MMI). By incorporating several diverse datasets, the robustness of the model is strengthened and can support broader generalizability across populations and conditions. It is important to note, however, that each of the datasets are largely collected in controlled or semi-controlled environments and contain mostly posed or elicited facial expressions. As this project applied this model to videos of children engaged in naturalistic gameplay, the emotional expressions were more subtle, spontaneous, and context dependent. As such, a degree of domain mismatch exists, and the model-derived estimates may contain classification errors, impacting the accuracy of emotion classification. Future research should incorporate ecologically valid data for training to improve the generalizability of emotion detection in educational environments.

Future research may build upon these findings by examining emotional dynamics across a broader range and number of digital games, including a wider array of mathematical concepts to enhance generalizability. Replication studies of this work, including a more diverse sample and larger sample size, are necessary to generalize the findings of this project. This work would benefit from a longitudinal design for a deeper understanding of the emotional regulation skill development and how this influences math learning. Future research should also assess the integration of real-time adaptive interventions specifically directed at lowering anger during digital learning. Further, developing systems that can respond to students’ dynamic emotional states would provide information on how real-time adaptive systems can measure and intervene for anger mitigation during digital gameplay, supporting subsequent math performance and learning. Finally, future research studies should be designed such that specific individual differences are assessed (i.e., baseline math anxiety) to understand which students may be more susceptible to the impacts of negative affect on learning and performance.

## 6. Conclusions

This study aimed to understand the relationship between real-time emotions, physiology, and performance in digital game-based learning environments, particularly within the context of mathematics. Previous research has relied on retrospective self-report measures to capture students’ affective experiences, while the present study implemented a real-time, multimodal approach to capturing and understanding students’ emotional experiences during digital gameplay. The findings demonstrate that students had lower performance on math-based games, as compared to visually and structurally identical non-math games. This result highlights the additional cognitive demands of math problem solving activities. Anger, as measured in real-time, emerged as a significant predictor of performance, specifically within math games, whereas other emotions and physiological arousal were not significant predictors. The results highlight that anger may play a significant role in shaping academic achievement in digital environments. Overall, this project contributes to the growing literature on affective experiences in digital learning environments by providing a novel ML approach and highlighting the importance of discrete emotional experiences, particularly anger, in shaping academic performance. The findings emphasize the need for educational technologies that support both cognitive processes and dynamic affective experiences that occur during learning.

## Figures and Tables

**Table 1:** Action Unit (AU) and descriptors for emotion classification.

Emotion	AU(s) Activated	Description of AU Activation
Happiness	AU6, AU12	AU6: Cheek raiser; AU12: Lip corner puller
Sadness	AU1, AU4, AU15	AU1: Inner brow raiser; AU4: Brow lowerer; AU15: Lip corner depressor
Anger	AU4, AU5, AU7, AU23	AU4: Brow lowerer; AU5: Upper lid raiser; AU7: Lid tightener; AU23: Lip tighten
Surprise	AU1, AU2, AU5, AU26	AU1: Inner brow raiser; AU2: Outer brow raiser; AU5: Upper lid raiser; AU26: Jaw drop
Fear	AU1, AU2, AU4, AU5, AU20, AU26	AU1: Inner brow raiser; AU2: Outer brow raiser; AU4: Brow lowerer; AU5: Upper lid raiser; AU20: Lip stretcher; AU26: Jaw drop
Disgust	AU9, AU15, AU16	AU9: Nose wrinkler; AU15: Lip corner depressor; AU16: Lower lip depressor
Contempt	AU12, AU14	AU12: Lip corner puller (asymmetrical); AU14: Dimpler

*Note*: AU numbers correspond to the Facial Action Coding System (FACS).

**Table 2:** Fixed effects of model.

Predictor	Estimate (*b*)	*SE*	*t*	*p*	Lower 95% CI	Upper 95% CI
Intercept	57.998	1.896	30.58	<0.001	54.281	61.715
Game Type (No Math)	26.801	2.523	10.62	<0.001	21.856	31.745
Heart Rate	1.840	1.382	1.33	0.185	−0.869	4.549
Anger	−15.245	5.674	−2.69	0.008	−26.367	−4.123
Disgust	−4.749	30.336	−0.16	0.874	−64.207	54.709
Fear	−10.472	23.750	−0.44	0.659	−57.021	36.077
Happiness	22.998	21.665	1.06	0.291	−19.466	65.461
Sadness	3.302	4.850	0.68	0.497	−6.204	12.809
Surprise	4.404	8.381	0.53	0.597	−12.023	20.831
AEQ Enjoyment	0.302	2.740	0.11	0.912	−5.068	5.672
AEQ Anxiety	0.060	2.408	0.03	0.978	−4.661	4.780
AEQ Boredom	−0.429	2.462	−0.17	0.864	−5.255	4.397

*Note:* Outcome performance listed per-game. Random intercepts were included for subjects. The 95% confidence intervals computed as β ± 1.96·*SE*. Random intercept *SD* for subject = 4.66; residual *SD* = 13.04. Observations = 128; subjects = 32.

**Table 3:** Model summary.

Group	Effect	Variance	*SD*
Subject	Intercept	21.79	4.66
Residual	Residual	170.00	13.40

*Note:* *N* = 32.

## Data Availability

All data can be made available upon request by emailing the corresponding author (ana.zdravkovic@mail.utoronto.ca).
